# Mental-health before and during the COVID-19 pandemic in adults with neurodevelopmental disorders

**DOI:** 10.1016/j.jpsychires.2023.01.029

**Published:** 2023-03

**Authors:** Amy Shakeshaft, Rachel Blakey, Alex S.F. Kwong, Lucy Riglin, George Davey Smith, Evie Stergiakouli, Kate Tilling, Anita Thapar

**Affiliations:** a Division of Psychological Medicine and Clinical Neurosciences, Centre for Neuropsychiatric Genetics and Genomics, Cardiff University, UK; b Wolfson Centre for Young People's Mental Health, Cardiff University, UK; c Population Health Sciences and MRC Integrative Epidemiology Unit, University of Bristol, Bristol, UK; d Division of Psychiatry, University of Edinburgh, UK

**Keywords:** ALSPAC, Neurodevelopmental disorders, COVID-19, Mental-health, ADHD, ASD

## Abstract

The COVID-19 pandemic negatively impacted mental health globally. Individuals with neurodevelopmental disorders (NDDs), including autism spectrum disorder (ASD) and attention deficit hyperactivity disorder (ADHD), are at elevated risk of mental health difficulties. We investigated the impact of the pandemic on anxiety, depression and mental wellbeing in adults with NDDs using data from the Avon Longitudinal Study of Parents and Children (n = 3058). Mental health data were collected pre-pandemic (age 21–25) and at three timepoints during the pandemic (ages 27–28) using the Short Mood and Feelings Questionnaire, Generalized Anxiety Disorder Assessment-7, and Warwick Edinburgh Mental Wellbeing Scale. ADHD and ASD were defined using validated cut-points of the Strengths and Difficulties Questionnaire and Autism Spectrum Quotient, self-reported at age 25. We used multi-level mixed-effects models to investigate changes in mental health in those with elevated ADHD/ASD traits compared to those without. Prevalences of depression, anxiety and poor mental wellbeing were higher at all timepoints (pre-pandemic and during pandemic) in those with ADHD and ASD compared to those without. Anxiety increased to a greater extent in those with ADHD (β = 0.8 [0.2,1.4], p = 0.01) and ASD (β = 1.2 [-0.1,2.5], p = 0.07), while depression symptoms decreased, particularly in females with ASD (β = −3.1 [-4.6,-1.5], p = 0.0001). On average, mental wellbeing decreased in all, but to a lesser extent in those with ADHD (β = 1.3 [0.2,2.5], p = 0.03) and females with ASD (β = 3.0 [0.2,5.9], p = 0.04). To conclude, anxiety disproportionately increased in adults with NDDs during the pandemic, however, the related lockdowns may have provided a protective environment for depressive symptoms in the same individuals.

## 1 Introduction

The COVID-19 (SARS-CoV-2) pandemic has caused widespread disruption to lives worldwide. Virus suppression measures were introduced in many countries, with restrictions including orders to stay at home; school, workplace and business closures; and limited social mixing. The impact of the COVID-19 pandemic on mental health is now a widespread concern (Holmes et al., 2020), with a number of studies indicating substantial increases in mental ill-health (Robinson et al., 2022), particularly elevated anxiety, loneliness and psychological distress, and decreased life satisfaction and mental wellbeing throughout the pandemic (Kwong et al., 2020; Niedzwiedz et al., 2021; Patel et al., 2022; Santomauro et al., 2021). Despite the greater risk of severe physical illness from COVID-19 existing for older people (Pijls et al., 2021), population studies have highlighted the disproportionate effect of the pandemic on the mental health of younger people (Lewis et al., 2022; McGinty et al., 2020a, 2020b; Niedzwiedz et al., 2021; Santomauro et al., 2021), likely influenced by job insecurity, financial pressures and education disruptions (Douglas et al., 2020; Gustafsson, 2020; Taylor et al., 2022).

Some groups have been identified as especially vulnerable to poor mental health during the pandemic; for example, those from the poorest households (Pierre et al., 2021) and those with a history of mental health problems (Bal et al., 2021; Kwong et al., 2020; Pierce et al., 2021; Warne et al., 2021). Another potential risk group are individuals with neurodevelopmental disorders (NDDs), such as attention deficit hyperactivity disorder (ADHD) and autistic individuals, who already have an elevated likelihood of mental health difficulties such as anxiety and depression (Faraone et al., 2021; Griffiths et al., 2019; Riglin et al., 2021b), as well as difficulties with work, education and social relationships (Farley et al., 2018; Thapar and Cooper, 2016) and thus may have fared especially poorly across pandemic restrictions.

Cross-sectional studies suggest this may be the case. One mixed-method cross-sectional study indicated higher rates of depression and anxiety symptoms in autistic adults compared to non-autistic individuals during initial pandemic restrictions (Oomen et al., 2021) and suggested that loss of routine, loss of social contact, and the anticipation of life going back to normal were the most difficult and stressful changes for autistic adults. However, in the same study, some reported the reduced sensory and social overload during restrictions were a positive factor. Studies of children and adolescents during the pandemic show that those with NDDs or special educational needs had the highest levels of mental ill-health (Creswell et al., 2021), with the majority of autistic children experiencing either a worsening of their pre-pandemic psychiatric diagnoses and/or the development of new psychiatric symptoms (Vasa et al., 2021). Whilst longitudinal studies in children and adolescents have indicated worse mental health in those with NDDs, the limited studies in adults have lacked pre-pandemic baseline assessments (Lewis et al., 2022), did not use longitudinal study designs or did not have repeated measurements during the pandemic (Kwong et al., 2020).

In this study, we expand on previous work by Kwong et al. (2020) which investigated mental health and factors relating to poor mental health during the early COVID-19 pandemic in UK longitudinal birth cohorts, by using the same cohort to investigate mental health in the subset of individuals with elevated NDD traits, specifically autism spectrum disorder (ASD) and ADHD, during three timepoints of the COVID-19 pandemic, comparing both to pre-pandemic levels, and to individuals without NDDs from the general population. Additionally, we investigate whether potentially influential circumstances during the pandemic, such as financial issues, family illness or COVID-19 infection, occurred as commonly in adults with NDDs as those without.

## 2 Methods

### 2.1 Sample

**Fig. 1:**
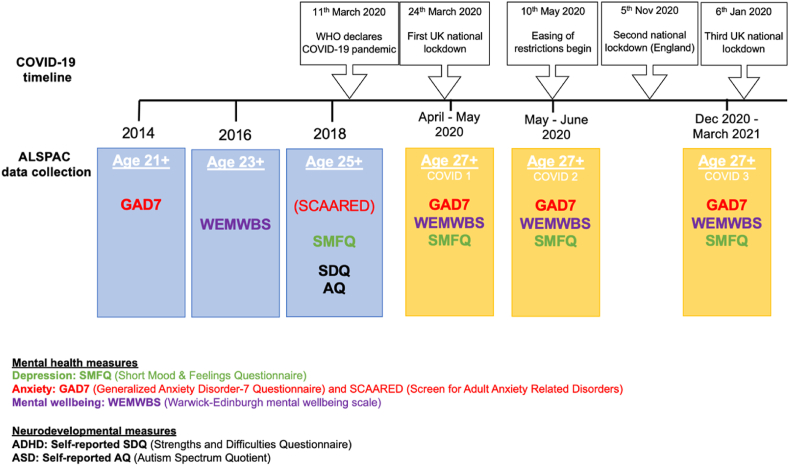
- Timeline of ALSPAC data collection relating to mental health and major COVID-19 events in the UK. World Health Organization (WHO).

The Avon Longitudinal Study of Parents and Children (ALSPAC) study is an ongoing longitudinal study which recruited pregnant woman in the Avon region of South-West England, due to give birth between April 1, 1991 and December 31, 1992 (Boyd et al., 2013; Fraser et al., 2013; Northstone et al., 2019). The core sample consisted of 14,541 mothers and of these pregnancies, 13,988 children were alive at 1 year. Following the initial recruitment, an additional 913 children were recruited in three phases. Data were collected from families repeatedly, including during the COVID-19 pandemic (Fig. 1). Full details of the ALSPAC study and sample can be found in the Supplementary Material. For families with multiple births, we included the oldest sibling. We analyzed data collected at ages 21, 23, 25 (pre-pandemic) and 27–28 (during pandemic). For further information on the cohort, including demographics see references Boyd et al. (2013); Fraser et al. (2013); Northstone et al. (2019).

### 2.2 Measures and data collection

#### 2.2.1 Pre-pandemic mental health

Anxiety symptoms were measured at age 21 using the self-rated Generalized Anxiety Disorder Assessment-7 (GAD-7) (Spitzer et al., 2006) (seven-items, range 0–21, higher values indicate more generalized anxiety disorder symptoms). Mental wellbeing was measured at age 23 using the self-rated Warwick-Edinburgh Mental Wellbeing Scale (WEMWBS) (Tennant et al., 2007) (14-item questionnaire, range 14–70, lower values indicate poorer mental wellbeing). Depressive symptoms were measured at age 25 using the self-rated Short Mood and Feelings Questionnaire (SMFQ) (Angold et al., 1995) (13-items, range 0–26, higher values indicate more depressive symptoms). Total symptom scores were calculated for each scale to generate a continuous measure. Each of these questionnaires has previously been used in ADHD and/or autistic populations (SMFQ (Hosozawa et al., 2021; Jarbin et al., 2020; Mazefsky et al., 2014), GAD-7 (Hull et al., 2021; Swansburg et al., 2021), WEMWBS (Hull et al., 2019b)).

We also used recommended cut-points for these measures to estimate the number of individuals in the dataset with probable depression (SMFQ ≥12) (Eyre et al., 2021), generalized anxiety disorder (GAD-7 ≥10) (Spitzer et al., 2006), and poor mental wellbeing (WEMWBS ≤40) (Warwick Medical School, 2021). These cut-points have previously been validated using area under the curve (AUC) analysis, with optimal sensitivity and specificity values chosen to detect those with depression (Bianco, 2012; Eyre et al., 2021; Warwick Medical School, 2021) and anxiety diagnoses (Spitzer et al., 2006).

#### 2.2.2 Adult ADHD traits

To capture those with elevated ADHD traits, we used the self-rated 5-item Strengths and Difficulties Questionnaire (SDQ) (Goodman, 1997) ADHD subscale. This was completed by ALSPAC participants at age 25. This is designed to measure hyperactive and inattentive symptoms, ranges from 0 to 10, and has been validated against a DSM-5 diagnosis of ADHD (Riglin et al., 2021a), with a cut-point of ≥6 recommended to capture approximately 10% of the sample. For conciseness, we refer to those with elevated ADHD traits (above the cut-point) as ADHD, however we acknowledge that this is not necessarily akin to an ADHD diagnosis. Questionnaires with >2 items missing were excluded. Details of imputation of missing items is in Supplementary Material. Additional sensitivity analysis of SDQ ≥5 (recommended for high validity in correctly identifying those meeting diagnostic criteria in young adulthood) and ≥7 (recommended for use in younger ages) cut-points (Riglin et al., 2021a) are reported in Supplementary Material.

#### 2.2.3 Adult autistic traits

To capture those with elevated autistic traits we used the self-rated Autism Spectrum Quotient (AQ). The AQ has been validated as a measure of clinical autism in adults, and ranges from 0 to 50, with a score of ≥32 indicating likely ASD (Baron-Cohen et al., 2001). This was completed by ALSPAC participants at age 25. For conciseness, we refer to those with elevated autism traits (above the cut-point) as ASD, however we acknowledge that this is not necessarily akin to an ASD diagnosis. Questionnaires with >10% (>5) missing items were not used. Details of imputation of missing items is in Supplementary Material. Additional sensitivity analysis of a broader cut-off point of AQ, suggested for screening of ASD (AQ ≥26 (Baron-Cohen et al., 2001)), is reported in Supplementary Material.

#### 2.2.4 COVID-19 mental health

Questionnaires were sent to participants during (i) the first national lockdown in the UK from April to May 2020, (ii) when restrictions eased from May to July 2020 and (iii) during another national lockdown from December 2020 to January 2021 (Fig. 1). For specifics of UK restrictions see https://www.instituteforgovernment.org.uk/charts/uk-government-coronavirus-lockdowns. During these three questionnaire periods, all at ages 27–28 (referred to as COVID1, COVID2 and COVID3), depression was measured using the SMFQ, anxiety using the GAD-7 and mental wellbeing using the WEMWBS. Additionally, the presence or absence of potentially influential events since the start of the pandemic (March 2020), namely loss of job; financial difficulties; illness/injury of someone close to participant; or death of someone close to participant were recorded at the COVID3 timepoint.

#### 2.2.5 COVID-19 infection

In addition to investigating mental health outcomes, we also used self-reported information recorded at the COVID3 timepoint on suspected or confirmed COVID-19 infections since the start of the pandemic, and the approximate date of infection, to investigate whether there were different distributions of infection in individuals with elevated ASD/ADHD traits.

### 2.3 Statistical analysis procedure

All analyses were conducted in R (R Core Team, 2021).

#### 2.3.1 Mental health during COVID-19

The primary sample for analysis of mental health consisted of those with complete neurodevelopmental (AQ & SDQ), sex and COVID-19 event data (detailed above) and mental health outcome data from at least one timepoint (pre-pandemic, COVID1, COVID2 or COVID3). Previous studies have investigated the potential bias from attrition in the ALSPAC cohort with regards to mental health during the pandemic with complete case analysis showing near identical results to those using an imputed sample (Kwong et al., 2020), therefore motivating our sample choice. The number of individuals and details of attrition are presented in Supplementary Figure 1. We initially describe cohort information, testing any differences between those with elevated vs. low ADHD/ASD traits using Chi-squared tests. We then present the prevalence of probable depression, generalized anxiety disorder and low mental wellbeing at pre-pandemic and COVID timepoints, stratified by those with ADHD/ASD. We also examined loss of job; financial difficulties; illness/injury of someone close to participant; or death of someone close to participant during the pandemic, and whether the frequency of these were different in those with ASD/ADHD compared to those without, since previous work has indicated similar factors influenced mental health during the pandemic in this cohort (Kwong et al., 2020).

To investigate whether those with elevated ADHD or ASD traits were differentially impacted by the pandemic, continuous mental health measures (SMFQ, GAD-7, WEMWBS) were modelled using multi-level mixed effects models using R2MLwiN (Zhang et al., 2016). These models account for correlations between repeated measures in the same individual and allow for different sample sizes at each timepoint, allowing maximization of the possible sample without imputing missing outcome data. Three models were run for each mental health outcome: 1) with timepoints and ADHD/ASD status included as fixed-effects; 2) sex-stratified models with timepoint and ADHD/ASD status included and 3) models including timepoint, ADHD/ASD status and sex with all possible interaction terms (2-way and 3-way) included. For all models, timepoints were included as dummy variables with the pre-pandemic timepoint as reference, ADHD or ASD traits were included as binary exposures based on cut-off points described above with low symptoms as reference, and sex was included with males as reference.

Supplementary analysis of the effect of ADHD-ASD comorbidity on mental health during the COVID-19 pandemic was undertaken by including both ASD and ADHD status and a three-way timepoint*ADHD*ASD interaction variable (and all intermediate interactions) in multi-level models of each outcome (for further details see Supplementary Material).

#### 2.3.2 Sensitivity analysis

As there was an average of 6 years difference between pre-pandemic and pandemic GAD-7 measurements, we tested for correlation between GAD-7 at age 21 and an additional measure of anxiety, Screen for Adult Anxiety Related Disorders (SCAARED) (Angulo et al., 2017), at age 25, to ensure anxiety at age 21 was related with anxiety at age 25, just prior to the pandemic. We also repeated analysis of mental health outcomes using a variety of different cut-off points for ADHD and ASD, to ensure broader and narrower classifications showed the same pattern of results. These are reported in the Supplementary Material. We also describe changes in the mean scores for individual items in the GAD-7 and SMFQ questionnaires in those with elevated vs. low ADHD and ASD traits.

#### 2.3.3 COVID-19 infection

Since previous work has indicated that individuals with ADHD and ASD may have been at increased risk of COVID-19 infection in the early stages of the pandemic (Das-Munshi et al., 2021; Liu et al., 2021; Merzon et al., 2021a, 2021b; Schott et al., 2021; Wang et al., 2021), and that, in ALSPAC study participants, COVID-19 infection was associated with worse mental health in the early stages of the pandemic (Kwong et al., 2020), we performed a secondary analysis of whether individuals with NDDs showed a different distribution of COVID-19 infection than those without. For this analysis, the sample included those with complete data on COVID-19 infection at COVID3 timepoint and complete ASD/ADHD exposure information. We first performed Chi-squared tests to determine whether the frequency of a suspected or confirmed COVID-19 infection by COVID3 timepoint was different in those with and without ASD or ADHD (as defined using the cut-offs described above). We then performed survival analysis using Kaplan-Meier Estimation (Kaplan and Meier, 1958) and performed a log-rank test (Mantel, 1966) to test for differences in COVID-19 infection distribution between those with elevated vs. low ADHD/ASD traits. The start date for survival analysis was set as November 17, 2019 for all cases, estimated to be the first date of COVID-19 infection by Roberts et al. (2021). The date of COVID-19 infection was the event date and individuals were right-censored if they had not experienced an infection by the date of COVID3 survey completion.

## 3 Results

### 3.1 Description of sample before and during the COVID-19 pandemic

Data on anxiety, depression and mental wellbeing from at least one appropriate timepoint (pre-pandemic, COVID1, COVID2, COVID3) alongside complete neurodevelopmental data were available from 3058 individuals (Supplementary Figure 1). Table 1 shows sample information. 386 (13%) individuals were classified with elevated ADHD traits at age 25, and 79 (3%) with elevated ASD traits. There was a higher proportion of males with elevated ADHD traits (males: 15%, females: 11%, p = 0.003) compared to females, however no difference in the proportion with elevated ASD traits (males: 3%, females: 3%, p = 0.84). 33 individuals (1%) had both elevated ADHD and ASD traits. Individuals with broadly defined ADHD were more likely to have experienced financial difficulties (OR = 1.34 [1.06,1.69], p = 0.01) and the death of someone close (OR = 1.33 [1.02,1.72], p = 0.03) during the pandemic (between March 2020 and March 2021).

**Table 1:** – Description of total sample and those with elevated vs. low ADHD and ASD traits, with p-values from Chi-squared tests. Note for the percentages of individuals above anxiety (GAD-7), depression (SMFQ) and poor mental wellbeing (WEMWBS) cut-points, percentages do not include missing data (for proportions see Supplementary Table 1).

	Total sample	Binary ADHD classification (SDQ ≥6)			Binary ASD classification (AQ ≥ 32)		
		Elevated traits	Low traits	P-value	Elevated traits	Low traits	P-value
**N**	3058 (100%)	386 (13%)	2672 (87%)	–	79 (3%)	2979 (97%)	–
**Sex**							
Females (%)	2103 (69%)	240 (62%)	1863 (70%)	**0.003**	53 (67%)	2050 (69%)	0.84
**ADHD traits**							
Mean SDQ score (SD)	3.04 (2.10)	*6.82 (0.99)*	*2.49 (1.60)*	*-*	4.82 (2.46)	2.99 (2.07)	–
ADHD based on SDQ ≥6 (%)	386 (13%)	*386 (100%)*	*0 (0%)*	*-*	33 (42%)	353 (12%)	**2.0x10^−10^**
**ASD traits**							
Mean AQ score (SD)	15.99 (7.19)	20.73 (7.97)	15.31 (6.80)	–	*35.61 (3.74)*	*15.47 (6.50)*	*-*
ASD based on AQ ≥ 32 (%)	79 (3%)	33 (9%)	46 (2%)	**2.0x10^−10^**	*79 (100%)*	*0 (0%)*	*-*
**Events during pandemic**							
Financial difficulties (%)	764 (25%)	116 (30%)	648 (24%)	**0.01**	23 (29%)	741 (25%)	0.39
Lost job (%)	239 (8%)	37 (10%)	202 (8%)	0.17	6 (8%)	233 (8%)	0.94
Death of someone close (%)	533 (17%)	82 (21%)	451 (17%)	**0.03**	10 (13%)	523 (18%)	0.26
Illness/injury of someone close (%)	514 (17%)	63 (16%)	451 (17%)	0.78	10 (13%)	504 (17%)	0.32
**Percentage above mental health cut-points**							
**Anxiety (GAD-7)**							
Pre-pandemic (%)	13%	21%	12%	**3.7x10^−5^**	26%	12%	**0.008**
COVID1 (%)	24%	37%	22%	**2.1x10^−7^**	42%	24%	**0.002**
COVID2 (%)	23%	41%	20%	**1.1x10^−12^**	46%	22%	**3.8x10^−5^**
COVID3 (%)	27%	42%	25%	**1.4x10^−12^**	47%	27%	**6.2x10^−5^**
**Depression (SMFQ)**							
Pre-pandemic (%)	21%	45%	17%	**1.8x10^−36^**	61%	20%	**8.2x10^−19^**
COVID1 (%)	15%	29%	13%	**8.0x10^−11^**	40%	14%	**1.3x10^−7^**
COVID2 (%)	18%	35%	16%	**2.6x10^−13^**	54%	17%	**1.9x10^−12^**
COVID3 (%)	19%	33%	16%	**3.8x10^−15^**	42%	18%	**5.1x10^−8^**
**Poor mental wellbeing (WEMWBS)**							
Pre-pandemic (%)	19%	34%	17%	**8.2x10^−12^**	42%	18%	**2.8x10^−6^**
COVID1 (%)	31%	47%	29%	**6.9x10^−9^**	61%	30%	**2.8x10^−6^**
COVID2 (%)	32%	50%	29%	**2.0x10^−10^**	60%	31%	**4.5x10^−6^**
COVID3 (%)	31%	49%	28%	**2.5x10^−17^**	60%	30%	**2.4x10^−8^**

“-” Indicates no statistical test was carried out.

The prevalence of anxiety, depression and poor mental wellbeing based on validated cut-off thresholds at each timepoint, are presented in Table 1. The proportion of those with ASD and ADHD with anxiety, depression and poor mental wellbeing were consistently higher than those without. The prevalence of anxiety in those with ADHD increased from 21% pre-pandemic to 42% by COVID3, and from 26% to 47% across the same timeframe in those with ASD. Depression prevalence fell from 45% to 33% in those with ADHD, and 61%–42% in those with ASD, while poor mental wellbeing increased from 34% to 49% in ADHD and 42%–60% in ASD.

### 3.2 Changes in mental health in ADHD and ASD during the COVID-19 pandemic

#### 3.2.1 ADHD

**Fig. 2:**
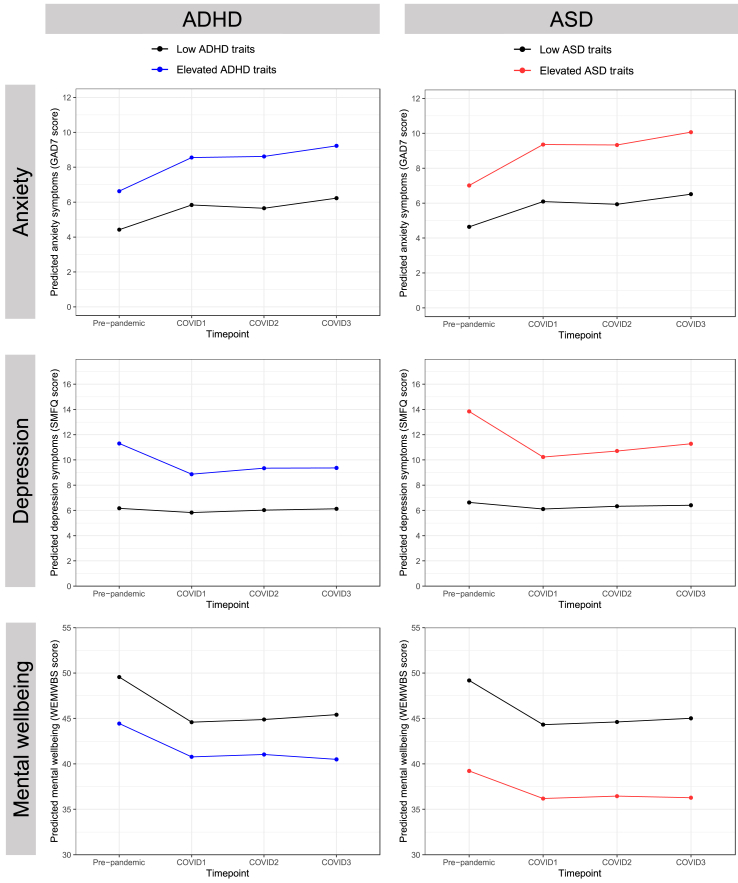
- Trajectories of anxiety, depression and mental wellbeing during COVID-19 pandemic in those with and without elevated ADHD/ASD traits. Shaded regions represent 95% confidence intervals.

**Fig. 3:**
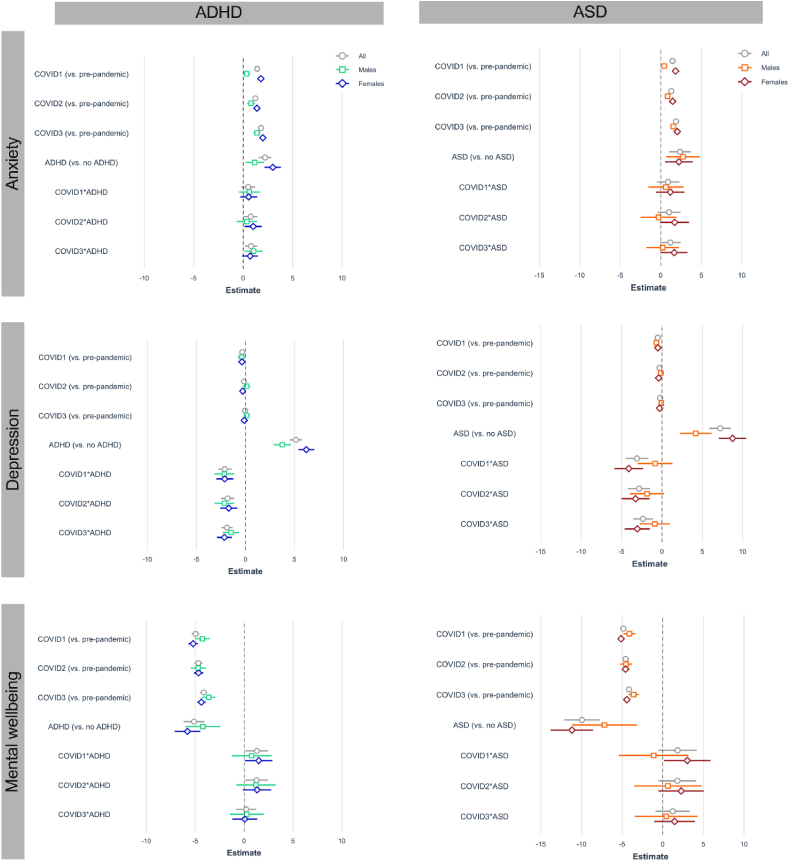
***-*** Results from multi-level models of mental health outcomes during the COVID-19 pandemic. Unstandardised β coefficient estimates with 95% confidence intervals from total and sex-stratified models are shown. P-values for estimates are presented in the Supplementary Material.

Anxiety symptoms were consistently higher in those with elevated ADHD traits compared to those without at all timepoints (Fig. 2). Anxiety was higher in those with and without ADHD at all COVID timepoints compared to pre-pandemic levels, but the increase in anxiety from the pre-pandemic level was elevated in those with ADHD at COVID2 (COVID2*ADHD β = 0.8 [0.1, 1.5], p = 0.03) and COVID3 (COVID3*ADHD β = 0.8 [0.2, 1.4], p = 0.01) timepoints (Fig. 3, Supplementary Table 2).

Depression symptoms were consistently higher in those with ADHD compared to those without (Fig. 2). During the pandemic, symptoms were lower than pre-pandemic levels in those with ADHD, whereas in those without ADHD, depressive symptoms stayed relatively stable. The decrease in depression between pre-pandemic and all 3 COVID timepoints was greater in those with ADHD than without (COVID1*ADHD β = −2.1 [−2.8,-1.4], p = 1.5 × 10^−9^; COVID2*ADHD β = −1.8 [−2.5,-1.1], p = 2.1 × 10^−7^; COVID3*ADHD β = −1.9 [−2.5,1.3], p = 1.5 × 10^−10^) (Fig. 3, Supplementary Table 4).

Mental wellbeing was consistently lower in those with ADHD compared to those without at each timepoint (Fig. 2), and was lower in those with and without ADHD at all COVID timepoints compared to pre-pandemic. The decrease in mental wellbeing from pre-pandemic levels was to a lesser extent in those with ADHD at COVID1 (COVID1*ADHD β = 1.3 [0.2, 2.5], p = 0.03) and COVID2 (COVID2*ADHD β = 1.3 [0.1, 2.4], p = 0.03) timepoints (Fig. 3, Supplementary Table 6).

A supplementary comparison of mean scores for each item of GAD-7 and SMFQ in those with and without ADHD are presented in Supplementary Figure 2 and 3. All GAD-7 items indicated worse anxiety symptoms during the pandemic compared to pre-pandemic in those with and without ADHD. For SMFQ, most items (8/13) indicated better (less) depressive symptoms during the pandemic. The item ‘felt no enjoyment’ indicated worse symptoms during the pandemic in both those with and without ADHD. Other items, such as ‘feeling miserable’, ‘feeling lonely’ and ‘finding it hard to think’ slightly improved on average in those with ADHD during the pandemic but got slightly worse in those without ADHD.

#### 3.2.2 ASD

The pattern of mental health trajectories among people with elevated ASD and elevated ADHD traits were similar. Anxiety symptoms were higher in those with ASD compared to those without at all timepoints, and higher in those with and without ASD during the pandemic compared to pre-pandemic levels (Fig. 2), showing some evidence of an elevated change compared to pre-pandemic levels in ASD (COVID3*ASD β = 1.2 [−0.1,2.5], p = 0.07) (Fig. 3, Supplementary Table 3).

Depression symptoms were consistently higher in those with ASD at each timepoint. During the pandemic, depression symptoms were lower than pre-pandemic levels in those with ASD, whereas in those without ASD, symptoms stayed relatively stable (Fig. 2). This decrease in depression between pre-pandemic and COVID timepoints was greater in those with ASD than without (COVID1*ASD β = −3.1 [−4.5,8.6], p = 1.6 × 10^−5^; COVID2*ASD β = −2.8 [−4.2,1.5], p = 4.9 × 10^−5^; COVID3*ASD β = −2.3 [−3.6,-1.1], p = 0.0002) (Fig. 3, Supplementary Table 5).

Mental wellbeing was consistently lower in those with ASD compared to those without, and was lower on average in both groups during the pandemic compared to pre-pandemic (Fig. 2). There was weak evidence that mental wellbeing decreased to a lesser extent from pre-pandemic levels in those with ASD compared to those without (COVID1*ASD β = 1.82 [−0.6,4.2], p = 0.13) (Fig. 3, Supplementary Table 7).

A supplementary comparison of mean scores for each item of GAD-7 and SMFQ in those with and without ASD are presented in Supplementary Figure 2 and 3. All GAD-7 items indicated worse anxiety symptoms during the pandemic compared to pre-pandemic in those with and without ASD. For SMFQ, most items (7/13) indicated better (less) depressive symptoms during the pandemic. The item ‘felt no enjoyment’ indicated worse symptoms during the pandemic in both those with and without ASD. Other items, such as ‘feeling miserable’, ‘feeling restless’, ‘feeling lonely’ and ‘finding it hard to think’ slightly improved on average in those with ASD during the pandemic but got slightly worse in those without ASD.

#### 3.2.3 Sex differences in mental health

Fig. 3 shows β coefficients from models stratified by sex as well as in the whole cohort, indicating the presence of sex differences in changes in anxiety, depression and mental wellbeing. The increase in anxiety at COVID2 and 3 is more pronounced in females with ASD (COVID2*ASD β = 1.7 [−0.6,3.9], p = 0.06; COVID3*ASD β = 1.7 [0.03,3.3], p = 0.05) compared to males with ASD (COVID2*ASD β = −0.3 [−2.5,1.9], p = 0.8; COVID3*ASD β = 0.2 [−1.8,2.2], p = 0.8). Depression decreases to a greater extent (greater improvement compared to pre-pandemic levels) and mental wellbeing decreases to a lesser extent (stays closer to pre-pandemic levels) in females with ASD at all three COVID timepoints. Inclusion of a 3-way interaction term in the model between COVID timepoints, ASD and sex (Supplementary Table 5) showed an effect modification of sex on the interaction between depression at COVID 1 and ASD, with females with ASD showing a greater decline in depressive symptoms compared to other strata (COVID1*ASD × Sex β = −3.3 [−6.3, −0.3], p = 0.03).

Sex-stratified mental health trajectories in those with and without ADHD and ASD are shown in Supplementary Figure 4. Females with ADHD/ASD had consistently the poorest mental health out of all strata at each timepoint investigated, and males without ADHD/ASD consistently had the best.

#### 3.2.4 ADHD and ASD comorbidity

Supplementary analysis of the effect of ADHD-ASD comorbidity showed that for all mental health trajectories (anxiety, depression and mental wellbeing) those with elevated ADHD and ASD traits (n = 33) had, on average, the poorest mental health at each timepoint (Supplementary Figure 5). These individuals also showed, on average, the greatest increase in anxiety and greatest decrease in depression symptoms from pre-pandemic to COVID timepoints (Supplementary Material).

#### 3.2.5 Sensitivity analysis

There was a large correlation between GAD-7 at age 21, and SCAARED at age 25 (Spearman's r = 0.54, p = 2.2 × 10^−16^). The effects of ADHD and ASD on changes in mental health remained very similar when using broader and narrower cut-off points for the SDQ and AQ (Supplementary Figures 6 and 7).

### 3.3 COVID-19 infection

Survival analysis did not show strong evidence of differences in the distribution of COVID-19 infection in those with ADHD (χ^2^ (1) = 1.1, p = 0.29) or ASD (χ^2^ (1) = 1.7, p = 0.19) compared to those without (Supplementary Material).

## 4 Discussion

In this study we investigate the impact of the COVID-19 pandemic on the mental health of young adults with NDDs. We show, on average, a substantial increase in anxiety during the pandemic among those with ADHD and ASD compared to those without. In contrast, mean depression symptoms improved in those with ADHD and in females with ASD, whilst remaining unchanged in those without. Mental wellbeing declined on average among all individuals, but this was to a lesser extent in those with ADHD and females with ASD.

It is commonly reported that individuals with NDDs such as ADHD and ASD are at an elevated likelihood to experience mental health difficulties, especially anxiety and depression, during the lifespan (Faraone et al., 2021; Griffiths et al., 2019; Riglin et al., 2021b). Results from this study indicate that the prevalence of anxiety, depression and poor mental wellbeing remain well above the population prevalence both before and during the pandemic, with 37–47% of individuals with elevated ADHD and ASD traits being classified as having a possible generalized anxiety disorder during the pandemic compared to between 20 and 27% of those without. The global increase in anxiety during the pandemic has attracted considerable concern, and here we show that this increase in anxiety was particularly marked for young adults with ASD and ADHD in the UK. Factors likely contributing to this increased anxiety include job instability and financial difficulties which were reported in around one-third of participants with ASD and ADHD compared to one-quarter of those without, as well as COVID-19 related distress (Adams et al., 2021). Previous work in this cohort has indicated that experiencing COVID-19 infection was associated with worse mental health outcomes during the early stages of the pandemic (Kwong et al., 2020), however, results from our survival analysis indicate that differences in mental health changes during the pandemic in those with ADHD and ASD were unlikely due to differing COVID-19 infections rates between groups.

In the case of depression, previous studies show little change in symptoms during the pandemic in the ALSPAC cohort (Kwong et al., 2020). This contrasts to evidence from a recent meta-analysis of longitudinal studies which showed an overall increase in depression and mood disorder symptoms during the pandemic (Robinson et al., 2022). However, data from our study indicates that, while the prevalence of depression remains higher in those with NDDs during the pandemic (up to 54% with ASD, and 35% with ADHD), depressive symptoms actually improved compared to pre-pandemic levels. For those with ADHD and females with ASD, depressive symptoms are the lowest during the first COVID timepoint, when the UK was in the strictest national lockdown, with schools and many workplaces closed and limitations in place on socializing and leaving home. Similarly, a longitudinal study in Western Australia showed that children and adolescents with ADHD reported decreases in depression and increases in positive wellbeing between pre-COVID and school closures due to the pandemic (Houghton et al., 2022). Qualitative studies in adults and children with ADHD have reported some positives of lockdown, including the flexibility of managing schedules, and less exposure to negative feedback (Ando et al., 2021; Bobo et al., 2020). Additionally, some autistic individuals reported that the relief of day-to-day social demands was appreciated during the early lockdown (Hedley et al., 2021), with less demands to camouflage or mask behavior (Bundy et al., 2022). This potentially protective environment of lockdown may be particularly prevalent for autistic females, for whom we saw the greatest decrease in depressive symptoms. Previous evidence indicates that autistic females commonly mask or camouflage behaviors in social situations in order to ‘fit in’ (Hull et al., 2017, 2019a) and camouflaging has been linked to greater symptoms of depression and anxiety in autistic adults (Hull et al., 2021). Therefore, it may be possible that the lack of expectation to socialize during the first COVID lockdown eased the pressure for females to camouflage autistic characteristics. Conversely, a qualitative study by Pellicano et al. (2021) indicated the enhanced social isolation accompanying the pandemic had a damaging impact on autistic individual's mental health and subjective wellbeing, including intensely missing friends and other social connections.

The opposite direction between changes in anxiety vs. depression symptoms in individuals with elevated ADHD/ASD traits was somewhat unexpected given the strong correlation between these two aspects of mental health and their known comorbidity (Johansson et al., 2013). However, evidence suggests that anxiety often precedes and can predict depression up to a decade later (Davies et al., 2016; Jacobson and Newman, 2014), and that anxiety changes more rapidly than depression (in the case of positive changes after treatment) (Lewis et al., 2019). Therefore, it may be too soon to predict the effect of the pandemic on long-term mental health outcomes. Future work with longer follow up data could use models with time lag effects to investigate the hypothesis that anxiety changes precede changes in depression after exposure to pandemic-related restrictions or other anxiety inducing events.

Whilst COVID-19 restrictions have mostly eased in the UK and many other countries at the time of writing, the results presented in this study, in addition to other studies, highlight the widespread effect the pandemic has had on mental health, particularly anxiety. This study indicates a disproportionate effect on anxiety in adults with NDDs which should be considered with regards to provision of support, both in the case of further restrictions and for management of mental health recovery in those most impacted (Vadivel et al., 2021). Future studies should aim to examine further changes in the mental health of autistic and ADHD populations as pandemic-related restrictions ease and, for most, socializing returns to normal, as well as factors contributing to resilience against and risk for mental ill-health in these populations.

This study has several important strengths. The longitudinal study design means robust pre-pandemic measures of mental health were present, preventing any chance of recall bias which may be present in cross-sectional or retrospective data collection. Also, multiple waves of data collection enabled us to capture mental health changes at different stages of national lockdowns. Further, by using multi-level models we were able to maximize the sample to include all available data.

The results of this study should be viewed considering methodological limitations. Like many longitudinal population-based studies, ALSPAC suffers from non-random attrition, with those at elevated risk of psychopathology, including those with high genetic risk for ADHD and depression, being more likely to drop-out (Taylor et al., 2018). Further, in this study we focused on ADHD and ASD traits in a population cohort, and although evidence suggests that both traits are continuously distributed (Larsson et al., 2012; Rutter and Pine, 2015; Thapar and Cooper, 2016), our findings may have limited generalizability to clinical samples of ADHD and ASD which have strict diagnostic criteria. The limited sample sizes for NDD groups, especially ASD (n = 79, 3%), is also a limitation, and contributes to wider confidence intervals for mental health estimates. The different timings of pre-pandemic measures of depression (age 25), mental wellbeing (age 23) and anxiety (age 21) are not ideal for assessing change from baseline, nor the 6-year gap between pre-pandemic and pandemic anxiety measures. However, we saw high correlation between anxiety scores at age 21 and at age 25 using SCAARED, indicating that this pre-pandemic measure is still acceptable for comparison. Further, we note that we cannot assess whether observed changes in mental health are a direct result of the pandemic rather than the natural trajectory of mental health, given the lack of comparison data on mental health trajectories in a non-pandemic exposed young adult population from this generation. Further, it should be noted that whilst the SMFQ, GAD-7 and WEMWBS are commonly used psychiatric assessment tools and have previously been used in NDD populations (Hosozawa et al., 2021; Hull et al., 2019b, 2021; Jarbin et al., 2020; Mazefsky et al., 2014; Swansburg et al., 2021), they have not specifically been validated in ASD or ADHD adult populations, and similarly, there is limited evidence on the ability of ADHD and ASD questionnaires (SDQ-E and AQ) in differentiating between ADHD/ASD symptoms and other psychiatric symptoms (i.e. Depression and anxiety) in adults (Radtke et al., 2019, 2019van Heijst et al., 2020). Our supplementary analysis of item-level responses for the SMFQ and GAD-7 should be interpreted with caution due to a lack of clarity of the reliability and validity of individual items in these scales, particularly in populations with high ASD/ADHD traits.

## 5 Conclusion

Prior to the COVID-19 pandemic individuals with NDDs were at heightened risk of poor mental health, but this study indicates this risk has substantially grown during the pandemic and associated lockdown measures. Risk factors for poor mental health including experiencing financial difficulties and death of someone close were also greater in those with NDDs during this period. Anxiety increased to a greater degree in young adults with elevated ASD/ADHD traits compared to those without, and mental wellbeing also decreased. The decline in depressive symptoms observed in individuals with ADHD and females with ASD raises issues surrounding the contribution of daily pressures such as camouflaging behaviors and maintaining strict routines to the development of poor mental health in adults with NDDs.

## Author contributions

Amy Shakeshaft: Methodology, Formal analysis, Investigation, Visualization, Writing – original draft, Writing – review & editing; Alex SF Kwong: Methodology, Investigation, Writing – review & editing; Rachel Blakey: Investigation, Methodology, Writing – review & editing; Lucy Riglin: Methodology, Conceptualization, Supervision, Writing – review & editing; George Davey Smith: Funding acquisition, Writing – review & editing; Kate Tilling: Conceptualization, Methodology, Funding acquisition, Project administration, Supervision, Writing – review & editing; Evie Stergiakouli: Conceptualization, Funding acquisition, Project administration, Supervision, Writing – review & editing; Anita Thapar: Conceptualization, Funding acquisition, Project administration, Supervision, Writing – review & editing

## Funding

The UK 10.13039/501100000265Medical Research Council and Wellcome (Grant ref: 217065/Z/19/Z) and the 10.13039/501100000883University of Bristol provide core support for ALSPAC. This publication is the work of the authors and AT and KT will serve as guarantors for the contents of this paper. This research was funded in whole, or in part, by the 10.13039/100010269Wellcome Trust (204895/Z/16/Z). For the purpose of Open Access, the author has applied a CC BY public copyright license to any Author Accepted Manuscript version arising from this submission. A comprehensive list of grants funding is available on the ALSPAC website (http://www.bristol.ac.uk/alspac/external/documents/grant-acknowledgements.pdf). ES, RB and KT work in a unit that receives funding from the 10.13039/501100000883University of Bristol and the UK 10.13039/501100000265Medical Research Council (MC_UU_00011/1 and MC_UU_00011/3).

## Declaration of competing interest

The authors report no conflicting interests.
